# Vertebrate Hedgehog signaling: cilia rule

**DOI:** 10.1186/1741-7007-8-102

**Published:** 2010-07-29

**Authors:** Christopher W Wilson, Didier YR Stainier

**Affiliations:** 1 Tumour Biology and Angiogenesis Department, Genentech Inc., 1 DNA Way, South San Francisco, CA, 94080, USA; 2 Department of Biochemistry and Biophysics, Program in Developmental and Stem Cell Biology, the Liver Center, Cardiovascular Research Institute and Diabetes Center, University of California, San Francisco, CA 94158, USA

## Abstract

The Hedgehog (Hh) signaling pathway differentially utilizes the primary cilium in mammals and fruit flies. Recent work, including a study in *BMC Biology*, demonstrates that Hh signals through the cilium in zebrafish, clarifying the evolution of Hh signal transduction.

See research article: http://www.biomedcentral.com/1741-7007/8/65

## 

Hedgehog (Hh) signaling is an evolutionarily conserved signal transduction pathway that is essential for a range of developmental patterning events, including specifying growth and polarity of the vertebrate limb and neural tube, and is misregulated in a number of cancers, for example basal cell carcinoma and medulloblastoma [1]. It has recently become clear that the primary cilium (Figure 1a), a non-motile microtubule-based structure that extends from membrane-docked basal bodies in most mammalian cell types, is essential for Hh signaling in the mouse, but not in the fruit fly *Drosophila melanogaster *[2]. Subsequent experiments with null mutants in components of the Hh pathway in zebrafish and mouse have raised questions about the conservation of the mechanisms of Hh signal transuction during evolution, and have suggested that utilization of the primary cilium for signaling might be a mammalian innovation. Now, data from several groups, including Kim *et al. * in *BMC Biology *[3], show that cilia are required for Hh signaling in zebrafish, revealing that their deployment in this pathway is thus not confined to mammals. Further, the studies demonstrate that the *iguana *gene product, originally implicated in the regulation of Hh signaling in zebrafish, has in fact a conserved role in ciliogenesis.

**Figure 1:**
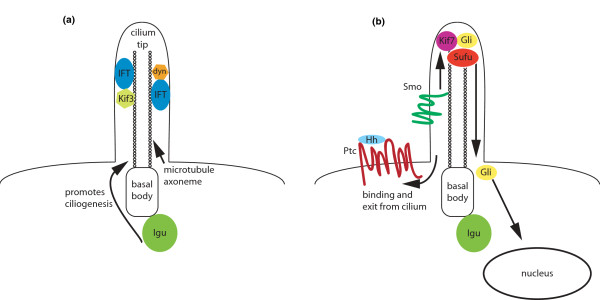
**Vertebrate Hh signaling is linked to the primary cilium**. **(a) **The primary cilium is composed of microtubule doublets extending from the basal body and proteins required for its construction include intraflagellar transport (IFT) proteins and Iguana (Igu). Kinesin-3 (Kif3) transports IFT particles towards the cilium tip, and dynein (dyn) returns IFT cargo to the base. **(b) **When Hh ligands bind to Patched (Ptc), the Hh-Ptc complex appears to exit the primary cilium, allowing translocation and activation of Smoothened (Smo). Smo signals to the Gli transcription factors, possibly through modulation of Kinesin-7 (Kif-7) and suppressor of Fused (Sufu). Gli proteins exit the cilium and enter the nucleus to promote transcription of Hh target genes after undergoing conversion to an activated state at an undefined point in this process.

## Hedgehog signaling and the primary cilium

Most of the important components of the Hh signal transduction pathway have been known for some time to be conserved from invertebrates to vertebrates [1]. The function of Hh is to regulate the activity of Gli-family transcriptional regulators (Ci in *Drosophila*, Gli in vertebrates). In the absence of Hh signaling, Ci/Gli transcriptional regulators undergo proteasome-dependent limited proteolysis to a truncated form in which they act as transcriptional repressors, a process that also requires the scaffolding protein Costal-2/Kinesin family member 7 (Cos-2/Kif7). Hh signaling causes Ci/Gli proteins instead to be converted, by an unknown mechanism, to transcriptional activators that translocate to the nucleus and activate target genes. This modification occurs as a consequence of the activity of the G-protein coupled-like receptor Smoothened (Smo), which in the absence of Hh is repressed by the Hh receptor Patched (Ptc). When secreted Hh binds Patched (Ptc), Ptc-mediated repression of Smo is disrupted and Ci/Gli's are activated, possibly through modulation of Cos-2/Kif7 conformation and localization.

A requirement for the cilium in this pathway was first suggested on the basis of ENU mutagenesis screens in mice, which showed that genes required for ciliogenesis are also required for Hh signal transduction (first reported by Kathryn Anderson's group and reviewed in [2]). Disruption of intraflagellar transport (IFT) genes, which encode components of a macromolecular machine that transports cargo on the ciliary axoneme, or of genes encoding the kinesin and dynein motors that transport the IFT complexes (Figure 1a) perturbs Hh signaling in the developing mouse embryo. In tissues that depend on Gli repressors for patterning, such as the limb bud, target genes are inappropriately activated in these mutants. Conversely, tissues that require high levels of Gli activation for patterning, such as the ventral neural tube, exhibit a loss of target gene expression. This combination of loss-and gain-of-function of Hh target genes is characteristic of most mutations that affect cilium formation or function.

Subsequent investigations with overexpressed and endogenous Hh pathway components showed that many are localized to the primary cilium (reviewed in [2]), and that Hh binding to Ptc appears to remove the Hh-Ptc complex from the cilium, permitting accumulation of Smo, Kif7, Gli2, and Gli3 along the ciliary axoneme or at the tip (Figure 1b). The dynamics of Gli transport into the cilium correlate well with transcriptional activation of Hh targets, leading to the hypothesis that Gli proteins are converted to activated forms in the cilium.

## Conserved utilization of primary cilia in zebrafish Hh signaling

Surprisingly, initial analysis in zebrafish suggested that cilia might not be involved in Hh signal transduction in this important model species, as *ift57*, *ift88 *and *ift172 *mutant embryos, in which intraflagellar transport is disrupted, do not exhibit overt loss-or gain-of-function Hh phenotypes. It now seems likely that this is due to the maternal contribution of transcripts and/or proteins for these genes, as zebrafish embryos lacking both maternal and zygotic *ift88 *function completely lack cilia and exhibit disrupted Hh signaling [4]. Kim and colleagues have addressed the same issue, by investigating the localization of Gli proteins in zebrafish using a GFP-tagged form of Gli2a in embryos injected with an engineered bacterial artificial chromosome (BAC) [3]. They were able to show that Gli2a-GFP restores the loss of Gli2a function in the zebrafish *you-too/gli2a *mutant, and localizes predominantly to the distal tip of the primary cilium [3]. Gli2a transport to the cilium is moreover controlled by the state of Hh pathway activation. Removal of the *ptc1 *and *ptc2 *genes, which creates a constitutively activated Hh signaling state, results in increased intensity of the Gli2a-GFP signal at the tip of the cilium compared with wild-type siblings. In a *smo *mutant background, where Hh signaling is abrogated, Gli2a-GFP signal intensity is reduced, although it is unclear whether this is due to a reduction in Gli2a-GFP protein levels or solely reflects dispersal of the signal along the length of the axoneme and the basal body. These data, with a prior report of Smo localization to primary cilia in zebrafish [5], indicate that in zebrafish, as in mouse, Hh signaling pathway components localize to the primary cilium and are transported along the ciliary axoneme in response to Hh signaling, and thus dependence of Hh signaling on the primary cilium is likely to be conserved across vertebrate lineages.

## Iguana/DZIP1: an ancient regulator of ciliogenesis

A second issue addressed by Kim and colleagues is the role of the *iguana *gene product in Hh signaling. The *iguana *(*igu*) locus was first identified as a modulator of Hh signaling in zebrafish, and found to encode a homolog of the human DAZ-Interacting Protein 1 (DZIP1), prominently expressed in embryonic stem cells and the germline [6,7]. Igu, which is a C2H2 zinc finger-coiled-coil domain protein, was originally proposed, on the basis of the mutant phenotypes and localization of an overexpressed Igu-GFP fusion protein, to regulate the nuclear-cytoplasmic shuttling of Gli proteins. However, the phenotypes of *iguana *mutants and morphants (morpholino injected embryos) [6,7] are extremely similar to those caused by a complete loss of Ift88 function mutation [4], which affects ciliogenesis; and Kim and colleagues [3] now show that rather than affecting nuclear transport of Gli2a-GFP, Igu is localized at ciliary basal bodies, in agreement with another recent report [8], and *igu *mutants have severely truncated primary ciliary axonemes, indicating effects on cilium formation [3]. On the basis of its localization, and the fact that it shares a coiled-coil domain with other ciliary proteins (such as Talpid3 and a subset of the IFT proteins), Kim *et al *propose that Igu may be an essential component of a multi-protein complex required for cilium formation [3].

This proposal is consistent with recent RNA interference studies in the planarian *Schmidtea mediterranea*, which is a focus of considerable interest because investigation of its regenerative capacity may lead to a better understanding of how regeneration is controlled. Knockdown studies of Igu and other ciliary proteins in planaria show that *igu *is required for ciliogenesis, though not for Hh signaling [9,10]. It is also in agreement with knockdown studies of human DZIP1 and DZIP1-like in immortalized retinal pigmented epithelial cells, which show disrupted primary cilia formation: as with zebrafish Igu, GFP fusions of these two proteins co-localized with the basal body [9]. Thus, Igu seems to be required for ciliogenesis throughout the metazoa, although there is so far no evidence for involvement of the primary cilium with Hh signaling outside of vertebrates.

## Open questions

Although there is a growing consensus on the conservation of the Hh signaling pathway, the role of the primary cilium, and how Igu connects the two, there is some disagreement on the part played by Igu in the construction of motile cilia. These specialized cilia contain an additional central pair of microtubules, as well as accessory dynein motors and macromolecular machines, and are not implicated in Hh signaling but have a critical role in the specification of left-right asymmetry (and thus organ laterality), and in fluid flow in the zebrafish kidney and brain. Motile cilia are found in Kupffer's vesicle in fish (where they specify left-right asymmetry), the ventral canal of the spinal cord and ependymal cells of the brain, and the pronephros. Kim *et al. * observed a reduction in the number of motile cilia in Kupffer's vesicle, but not in the pronephros. Others by contrast have found a reduction in cilia in both regions of the embryo, and varying effects on left-right asymmetry and kidney cyst formation [8,9]. Effects of *igu *deficiency on motile cilia may be secondary to its effects on Hh signaling, which is required for the expression of *foxj1*, a master regulator of the motile cilium formation program [3]. Further, the differing observations from various groups may be due to a redundant role for *igu*/*dzip1 *and *dzip1-like *[8,9], and/or differences between morphant and mutant phenotypes. Thus, further, meticulous analysis of *igu *and *dzip1-like *morphants and mutants will be necessary to resolve this issue.

The function of Igu in ciliogenesis appears to be conserved in most metazoa, but its role in Hh signaling has thus far been shown only in vertebrates. It is not yet known whether Hh signaling utilizes cilia in most organisms (and fruit flies have thus 'lost' the cilium-Hh connection), or if Hh signal transduction co-opted cilia later in evolution [9,10]. The ancient ciliogenic function of Igu provides an excellent entry point for the study of these two processes in other metazoans.
